# Surveying the endomicrobiome and ectomicrobiome of bark beetles: The case of *Dendroctonus simplex*

**DOI:** 10.1038/srep17190

**Published:** 2015-11-26

**Authors:** Audrey-Anne Durand, Amélie Bergeron, Philippe Constant, Jean-Philippe Buffet, Eric Déziel, Claude Guertin

**Affiliations:** 1 INRS-Institut Armand-Frappier, Laval, QC, H7V1B7, Canada

## Abstract

Many bark beetles belonging to the *Dendroctonus* genus carry bacterial and fungal microbiota, forming a symbiotic complex that helps the insect to colonize the subcortical environment of the host tree. However, the biodiversity of those bacteria at the surface of the cuticle or inside the body parts of bark beetles is not well established. The aim of this study was to characterize the bacterial microbiome associated with the eastern larch beetle, *Dendroctonus simplex*, using bacterial 16S rRNA gene pyrosequencing. The ecto- and endomicrobiome and the subcortical galleries were investigated. Several bacterial genera were identified, among which *Pseudomonas, Serratia* and *Yersinia* are associated with the surface of the beetle cuticle, and genera belonging to Enterobacteriaceae and Gammaproteobacteria with the interior of the insect body. The index of dissimilarity indicates that the bacterial microbiome associated with each environment constitutes exclusive groups. These results suggest the presence of distinct bacterial microbiota on the surface of the cuticle and the interior of *D. simplex* body. Additionally, the bacterial diversity identified in the galleries is substantially different from the ectomicrobiome, which could indicate a selection by the insect. This study reports for the first time the identification of the eastern larch beetle microbiome.

Symbiotic microorganisms are crucial for plant feeding insects: they are involved in the food digestion processes and provide supplemental nutrition and detoxification of plant defense compounds^1^,^2^,^3^. Several bark beetles belonging to the *Dendroctonus* genus carry a symbiotic complex that helps the insects to colonize their host trees^1^,^4^. Symbiotic bacteria supplement the phloem diet with amino acids^5^,^6^, vitamins^3^ and nitrogen^7^,^8^,^9^. For example, *Enterobacter agglomerans* and *Enterobacter* spp., which are associated with larvae and adults of *Dendroctonus terebrans*, are nitrogen-fixing bacteria^7^. Gammaproteobacteria and Actinobacteria isolated from *Dendroctonus rhizophagus* are capable of cellulose breakdown^10^. Furthermore, in response to the attacks of the beetles, coniferous trees secrete and release deterrent terpenes, which are toxic for phloeophagous insects^11^. Some *Pseudomonas* species isolated from *Dendroctonus ponderosae* and *Dendroctonus valens* are capable to metabolize the terpenoid molecules, which seems to facilitate the colonization of the host trees by the beetles^12^,^13^. The microorganisms may also be involved in immunity processes in some beetle species^14^. Accordingly, some bacteria of the symbiotic complex produce secondary metabolites, some with antifungal activities^15^,^16^,^17^. These symbiotic bacteria may protect the insects against entomopathogenic fungi used as biological control agents^17^,^18^. For example, *Streptomyces* isolated from *Dendroctonus frontalis* produces mycangimycin, an antifungal molecule that inhibits the growth of the antagonistic fungus *Ophiostomas minus*^17^.

The eastern larch beetle, *Dendroctonus simplex* LeConte (Coleoptera: Scolytinae), is a phloeophagous beetle for which the microbiome has not yet been characterized. This beetle is a secondary pest that usually attacks freshly dead or weakened trees. However, the eastern larch beetle may undergo epidemic outbreaks; in such cases they may attack healthy trees^19^,^20^. During the dispersal phase, the pioneer beetles bore holes in the tree trunk and subsequently buildup galleries into the phloem layer. Gallery construction causes severe desiccation, bark decay and eventually tree death^20^,^21^. The widespread outbreaks observed in the past suggest that the identification of these beetles as a secondary pest should be reconsidered^20^.

The identification of microbial communities associated with *Dendroctonus* species has been mainly performed by using culture-dependent methods or through targeted sequencing following gene cloning^10^,^22^. Even though fungi are crucial partner in insect’s microbiome, this paper attempts to focus on bacteria. Symbiotic bacteria have been isolated from the inside or the outside of the insect body. Mainly, ectosymbiotic bacteria are related to the mouthparts and cuticles of the insects while endosymbiotic bacteria are mostly found in the body cavity, in the gut and within cells^23^,^24^,^25^. More recently, high throughput sequencing and community metagenomic analyzes have been used to identify the microorganisms associated with bark beetles^13^,^26^. These approaches allow for a better resolution of the existing diversity in the samples. However, none of the previous studies investigated the difference that may exist between the ectomicrobiome and the endomicrobiome of an insect. Therefore, bacterial 16S rRNA gene pyrosequencing was used to identify bacteria associated with the eastern larch beetle. The aim of this research was to characterize the bacterial diversity associated with the surface of the cuticle, the interior of the insect body, as well as that found in subcortical galleries. Analyzes were performed to compare the bacterial diversity from the different microenvironments. We report the first comprehensive characterization of the bacterial diversity associated with the surface of the cuticle and the interior of *D. simplex*. Our results revealed non-random mutually exclusive bacterial communities, which demonstrate a specific organization between the three different environments.

## Materials and Methods

### Site location and sample preparation

Eastern larch beetles, along with a sample from their galleries, were collected from a Quebec provincial larch plantation located in St-Claude (Québec, Canada; Lat. 45.6809, Long. −71.9969) with the permission of the ministère des Forêts, de la Faune et des Parcs authority. To obtain pioneer beetles, newly attacked hybrid larch trees were selected and harvested, and logs were transported to the laboratory, just after the insect flight activity. The tree sections were kept overnight at 4 °C until the insects were harvested. Each *D. simplex* adult was picked using sterilized tweezers and placed into separated sterile 2 ml microcentrifuge tubes. The insects were recovered by gently peeling off the bark from the entrance holes until the insects were reached. A 1 cm section of the galleries was also collected near the insect entrance hole and placed in a sterile microcentrifuge tube.

The bacterial microbiome associated with the surface of the cuticle and the interior of the insect body, and the galleries were investigated. For each environment, three replicates were prepared. For each replicate, fifty insects were randomly selected and pooled in 15 ml polypropylene tubes to recover sufficient bacterial genomic DNA from the surface of the cuticle. Five consecutive washes in 5 ml phosphate-buffered saline (PBS) (137 mM NaCl, 2.7 mM KCl, 10 mM Na_2_HPO_4_, 2 mM KH_2_PO_4_, pH 7.4) containing 0.1% Triton X-100 with 1 min agitation (Fisher Vortex Genie 2, Ottawa, ON, Canada) were applied to each sample. The pooled microbial suspensions were then filtered through a 0.22 μm nitrocellulose filter (EMD Millipore, Billerica, MA, USA) to concentrate the biomass. Each filter was placed in a Lysing matrix A tube (MP Biomedicals, Solon, OH, USA) until DNA extraction.

For the endomicrobiome of *D. simplex*, ten adults among the 50 washed insects were randomly selected per replicate. The surface of the insects was sterilized with three serial washes in 1 ml 70% ethanol (EtOH) with 1 min vortexing. Finally, 1 ml sterile water was used to wash the remaining ethanol, and the insects were then carefully crushed in 200 μl PBS containing 0.1% Triton using sterilized mortar pestles fitting 1.5 ml microcentrifuge tubes (VWR, Mont-Royal, QC, Canada). For each sample, the homogenate was transferred into a 2 ml screw cap tube containing 200 mg of 0.1 mm glass beads (BioSpecs, Bartlesville, OK, USA). This material was used for DNA extraction.

The bacterial microbiome associated with the subcortical galleries was recovered from the galleries where the insects were collected. A total of 25 galleries were selected per replicate. First, insect frass was removed, and the inside galleries were carefully scraped using a sterile scalpel. For each selected galleries, the material was then placed in an individual sterile microtube. As previously described with ectomicrobiome of larch beetle, five washes and agitations with PBS-Triton X-100 solution were performed, followed by filtration to recover bacteria. Each filter was then transferred into a Lysing matrix A tube until DNA extraction.

### DNA extraction and PCR amplification

Total DNA was extracted using mechanical lysis method. Briefly, 1 ml extraction buffer (50 mM Tris-HCl, 5 mM EDTA-2Na, 3% SDS, pH 8.0) containing 20 μg/ml RNase A was added into tubes containing the ectobacteria, the endobacteria, and those from the corresponding galleries. Cell lysis was achieved using the FastPrep®-24 Instrument (MP Biomedicals, Solon, OH, USA). Two cycles of lysis at 4 m/s for 50 s followed by 5 min on the ice were performed. The tubes were then centrifuged at 16,800 × *g* for 5 min, and the supernatant recovered before the second lysis cycle. For each sample, ammonium acetate was added to the combined supernatants at a final concentration of 2 M. Tubes were briefly agitated by inversion and kept on ice for 5 min before centrifugation at 20,800 × *g* for 15 min at 4 °C. The supernatant was collected and maintained on ice for 5 min before a second centrifugation with the same parameters was done. Supernatants were collected and DNA precipitated overnight at 4 °C by adding an equal volume of isopropyl alcohol (2-Propanol). Centrifugation at 20,800 × *g* at 4 °C for 30 min was performed, and the supernatant discarded. Two DNA pellet washes with cold EtOH 70% and centrifugation at 20,800 × *g* for 15 min at 4 °C were finally done. The pellets were air-dried and suspended in sterile water. DNA concentration was estimated using the Quant-iT™ PicoGreen® dsDNA Assay Kit (Invitrogen, Life Technologies, Burlington, ON, Canada) following the manufacturer instruction.

In order to document the presence of bacterial DNA in each sample, the 16S rRNA gene was amplified by PCR using the universal primers pA-27-YM (5′ AGA GTT TGA TYM TGG CTC AG 3′)^27^ and pH (5′ AAG GAG GTG ATC CAR CCG CA 3′)^28^. All PCR reactions were carried out in a 50 μl volume containing 25 mM MgCl_2_, 10 μg BSA, 10 mM dNTPs, 10 mM of each primer, 5 U Taq DNA polymerase and 10× ThermoPol® buffer (New England Biolabs, Whitby, ON, Canada). Following the initial denaturation step of 5 min at 94 °C, 30 amplification cycles were performed (94 °C for 45 s, 55 °C for 45 s, 72 °C for 45 s) followed by a final extension step at 72 °C for 10 min. Amplification was confirmed by electrophoresis of the PCR products on a 1.5% agarose gel stained with ethidium bromide and visualized under UV light. A negative control containing all the extraction reactive but no insect was realized and no amplification was observed.

### 16S rRNA pyrosequencing

DNA samples were sequenced by Research and Testing Laboratory, LLC (Lubbock, TX, USA). PCR amplification of bacterial 16S rRNA gene was done using the universal primers 28F (5′ GAG TTT GAT CNT GGC TCA G 3′) and 519R (5′ GTN TTA CNG CGG CKG CTG 3′) covering the V1-V3 variable regions. The amplicons were pyrosequenced using Roche 454 Titanium chemistry. Elongation was performed from the forward primer. Raw data are available on NCBI under BioProject number PRJNA275539.

### Sequence processing pipeline

The post-sequencing processing were completed using the open-source program mothur v.1.33.0 software (http://www.mothur.org)^29^ following the pipeline described by Comeau *et al*.^30^. Raw 454 reads were first processed to remove low-quality reads, such as (i) the presence of one or more uncertain bases (N), (ii) sequences shorter than 150 nt (nucleotides), (iii) unusually long reads that extended more than 100 nt over the amplicon size, (iv) reads that had long homopolymers sequences (more than 8), and (v) reads that had incorrect forward primer sequences. Sequences corresponding to forward primers were kept to facilitate the alignment of the sequences during subsequent analyzes. Contaminants, such as chloroplasts and mitochondria, were eliminated before removing the chimeras with UCHIME^31^, as implemented in mothur. The remaining filtered sequences were aligned by domain against the provided SILVA reference alignment^32^ using the ksize = 9 parameter in mothur. Manual alignment was performed to correct the misaligned sequences with BioEdit 7.2.5 (http://www.mbio.ncsu.edu/bioedit/bioedit.html). Reads were also trimmed of all bases beyond the reverse primer. Singletons were finally removed after clustering into draft Operational Taxonomic Units (OTUs) to obtain the final quality reads. An equalization step was performed to obtain equal sampling depth for each sample. The final aligned reads were clustered into OTUs at ≥ 95% similarity level using the furthest neighboring cluster in mothur, thus allowing for the identification of the bacterial genus^33^. The measure of diversity and community similarity analysis were also obtained based on mothur analysis. Sequences were taxonomically identified using the Ribosomal Database Project (RDP) classifier version 2.6^34^ trained on 16S rRNA training set 9.

### Diversity analysis

Rarefaction curves were generated within the software mothur to evaluate the sufficiency of the sampling effort using equalized data (*i.e*. random selection of a determined number of sequences from each library according to the size of the smallest library to avoid bias due to variable sampling efforts). The abundance-based coverage estimator (Ace) and the Shannon index, also generated with mothur, were used to estimate the diversity among the bacterial populations using the same data.

To proceed with the diversity analyzes among the bacterial communities, a maximum likelihood phylogenetic tree was constructed with MEGA 5.2^35^ using a representative sequence of each previously generated OTU and an Archaea sequence as the outgroup. A sample ID mapping file was also built to indicate to which sample each OTU belongs as well as the number of times each sequence was observed (sequence abundance). Finally, a category-mapping file was constructed to relate the sample names in the sample ID mapping file to their related data. A cluster of similarity was generated with Fast UniFrac (http://bmf2.colorado.edu/fastunifrac/)^36^,^37^ to compare all communities simultaneously and to observe which communities are phylogenetically similar. Average agglomerative clustering (UPGMA) based on computed UniFrac distances and the abundance of each OTU was calculated using a Jackknife procedure with 1,000 permutations and 75% of the sequences to verify whether partitioning of ribotyping profiles corresponded to the three ecological niches surveyed. To visualize the bacterial community across all samples, a heat map was generated on the log-transformed abundance using the MultiExperiment Viewer (MeV) v4.9^38^. The representative sequence of each of the 90 OTUs found in abundance (≥1% of sample abundance), as well as their closely related sequences identified by BLASTN against NCBI database were aligned together with the MUSCLE^39^ algorithm implemented in MEGA. A maximum likelihood phylogenetic tree was built with FastTree 2.1.7^40^ using the GTR model with 1,000 resampling to estimate node support values. Additionally, a Venn diagram was also generated with mothur to observe the partition of the OTUs across the three environments sampled. Finally, a principal coordinate analysis (PCoA) was also achieved with Fast UniFrac to assess whether different bacterial communities were distributed along the axes of variation using the same previous reference files.

## Results

### Bacterial diversity associated with the eastern larch beetle microbiome

**Figure 1:**
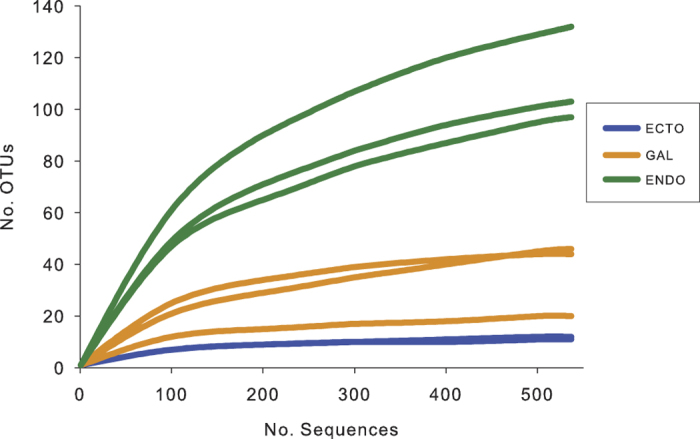
Rarefaction curves of OTUs diversity for each sample. Each of the samples contains 537 sequences to obtain equal sampling depth. “Ecto”, ectomicrobiome; “Gal”, microbiome of the galleries; “Endo”, endomicrobiome.

To investigate the complete bacterial microbiome associated with the eastern larch beetle, ecto- and endobacteria, as well as the bacteria located in the galleries were recovered. For each environment, three replicates were analyzed, except for the ectobacteria, where the quantity of bacterial genomic DNA extracted from one of the pooled samples was not sufficient to perform pyrosequencing. A total of 108,447 raw sequences were obtained, and 48,380 high-quality-filtered sequences were recovered. The average read length was 422 bp. Comparison of the diversities between the endo-, the ectomicrobiome and the microbiome of the galleries was performed on equalized-sequence number. Because one sample contained less sequences of quality than the other samples, 537 sequences were kept for each sample, representing a total of 4,296 sequences. After clustering at a 95% pairwise-identity threshold, 278 OTUs were recovered, from which only 26% enclosed a unique sequence. Fig. 1 shows the rarefaction curves associated with the eight samples. Asymptotes are reached for the samples coming from the ectomicrobiome and the microbiome of the galleries, suggesting that the OTUs observed are representative of the whole bacterial diversities. As for the endomicrobiome, rarefaction curves tend to reach a plateau, indicating that most of the diversity was recovered, but not all of it. The endomicrobiome displays a higher bacterial richness than the other two environments. Hence, among the OTUs containing only one representative sequence, 74% were associated with the endomicrobiome. Consequently, the ectomicrobiome displays the lower richness, with only 6 OTUs representing 95% of the diversity.

The abundance-based coverage estimator (Ace) and Shannon diversity index for each of the samples were computed (Table 1). The Shannon indices show a significant difference between the endo-, the ectomicrobiome and microbiome of galleries with values, respectively, of 4.15, 1.27 and 2.24 (ANOVA test; *F* = 60.14; *p <* 0.0003). On the other hand, for Ace estimator results, the environment seems to have a significant effect on the microbial richness (ANOVA test; *F* = 20.88; *p <* 0.0037). However, no difference was observed between the ectomicrobiome (16.68) and the microbiome of the galleries (60.93). Endomicrobiome (148.90) shows a significant difference in the bacterial richness found in the two other environments.

### Taxonomical composition and variability of bacterial communities associated with the eastern larch beetle

**Figure 2:**
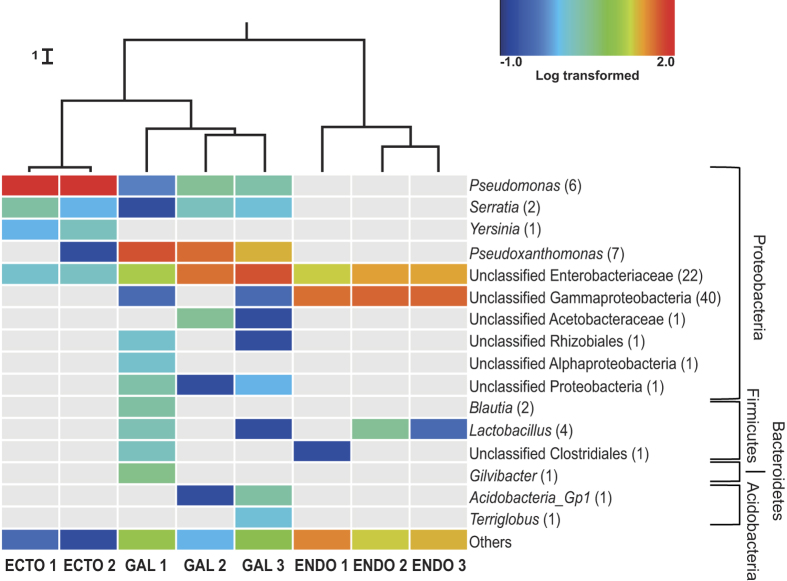
Bacterial community composition of the eastern larch beetle and the galleries. Only high quality filtered sequences were used to generate the heat map. Abundant OTUs (≥1% of the total abundance) are represented in the chart. The non-abundant OTUs (<1%) are grouped in the category named “others”. The heat map represents the log-transformed relative abundance and grey representing the absence of the OTU. Number in parenthesis represents the number of OTUs associated with each genus. Cluster of similarities regrouping the different samples is represented above the heat map, with each node supported by a Jackknife analysis with greater than 99.9% accuracy. “Ecto”, ectomicrobiome; “Gal”, microbiome of the galleries; “Endo”, endomicrobiome.

Each of the 278 OTUs was taxonomically identified using the RDP classifier. Most of the identified OTUs belong to the phyla Acidobacteria, Bacteroidetes, Firmicutes and Proteobacteria (Fig. 2). Within the Proteobacteria, Gammaproteobacteria were largely predominant, although a few Alphaproteobacteria OTUs were also identified. Only one predominant bacterial genus, *Pseudomonas*, was associated with the surface of the cuticle with 95% of the sequences total abundance. Two other genera, *Serratia* and *Yersinia*, were also considered abundant, representing respectively 2% and 1% of the sequences. Unclassified bacteria belonging to the Enterobacteriaceae family were also associated with the insect cuticle with about 2% of the total sequences found.

Unclassified Gammaproteobacteria are the most prevalent OTUs associated with the endomicrobiome, followed by bacteria from the Enterobacteriaceae family, which together represent approximately 70% of the total abundance. *Lactobacilli* were also found in two of the three samples. A great number of non-abundant bacteria were found in those samples, representing 20% to 35% of the diversity.

A higher number of abundant bacterial OTUs (≥1% of total abundance) were identified in the galleries samples then in the ecto- and endomicrobiome of *D. simplex*. For all replicates, bacterial OTUs belonging to the *Pseudoxanthomonas* genus and the Enterobacteriaceae family represent almost 80% of the total sequences abundance. Other genera were also identified, such as *Pseudomonas, Serratia, Acidobacteria* Gp1 and *Lactobacillus*, each of them representing more than 1% of the galleries relative abundance. In addition, unclassified bacteria belonging to several phyla were found in these samples. Between 2% to 10% of the OTUs were placed in the category “others”, depending on the replicate. Experiments were conducted for a second year, and results were consistent with previous identification.

A weighted UniFrac UPGMA dendrogram (Fig. 2) was computed to assess differences between microbial communities based on phylogenetic information. For each environment (ectomicrobiome, endomicrobiome and microbiome of the galleries), the different replicates clustered together, showing the consistency of the sequences obtained and the specificity of each environment. Additionally, the samples coming from the insect’s cuticle and the galleries are more closely related, while OTUs associated with the endomicrobiome of *D. simplex* form a more distinct phylogenetic group. A Jackknife analysis was performed to establish the robustness of each node of the environmental cluster. Each node of the dendrogram was recovered with more than 99.9% accuracy, showing the validity of the similarity clustering.

**Figure 3:**
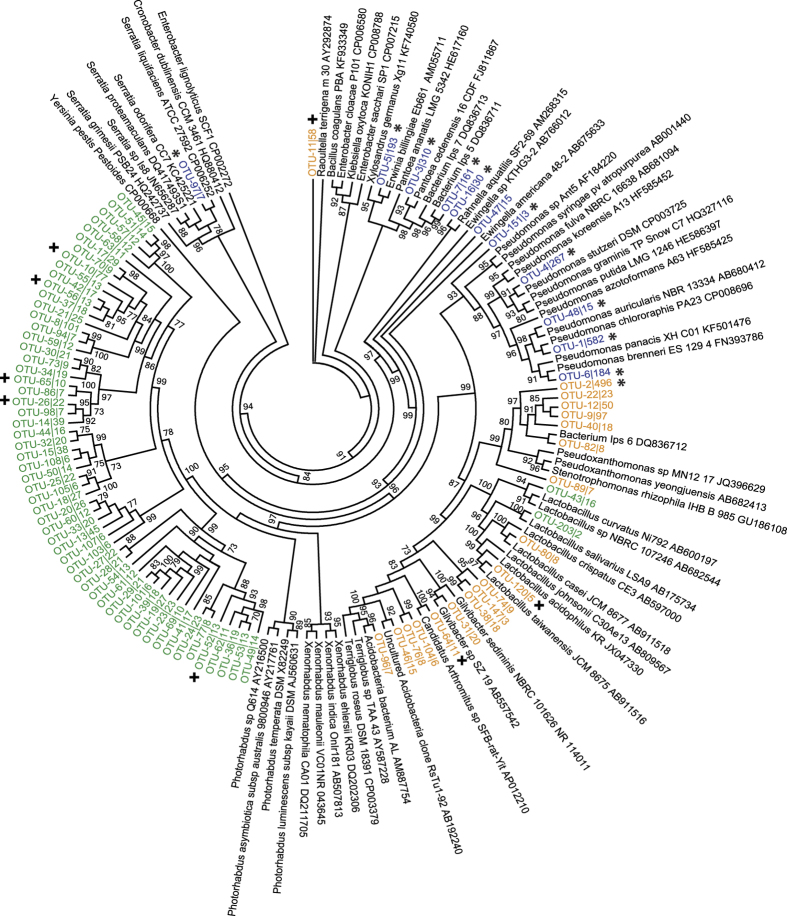
Phylogenetic affiliation of the abundant bacterial OTUs associated with the eastern larch beetle based on partial 16S rRNA gene sequences. Closely related sequences were identified using BLASTN against NCBI database. Sequences were aligned using the MUSCLE algorithm implemented in Geneious. Maximum phylogenetic tree was constructed with FastTree using the GTR model with 1,000 resampling. The colors used for the OTUs represent the environment they belong to (blue = ectomicrobiome, orange = microbiome of the galleries, green = endomicrobiome, * = presence in ectomicrobiome and galleries and + = presence in endomicrobiome and galleries).

To further investigate the taxonomic identification of the most abundant OTUs across the three ecological niches, a maximum likelihood phylogenetic tree was constructed using FastTree based on the representative 16S sequences of each OTU and their closely related sequences identified by BLASTN (Fig. 3). Phylogenetic analysis revealed taxa related to genera *Pseudomonas*, *Pantoea*, *Erwinia*, *Rahnella*, *Serratia* and *Ewingella* on the surface of the insect’s cuticle. Whereas, in the interior of the insect body, most OTUs (56) formed a well-delineated and monophyletic cluster representing potentially novel bacterial genera belonging to the family Enterobacteriaceae. Among the closest relatives of this cluster (85% identity) were *Photorhabdus* and *Xenorhabdus*. Likewise, for the endobacteria, two OTUs were affiliated to *Lactobacillus* (Firmicutes). Finally, the most frequently found OTUs in galleries exhibit phylogenetic relationships with *Pseudoxanthomonas*, *Gilvibacter*, *Terriglobus*, *Lactobacillus* and *Raoultella*. Furthermore, some OTUs highly abundant in the endomicrobiome have also been recovered from the galleries.

### Bacterial community comparison between sampled environments

**Figure 4:**
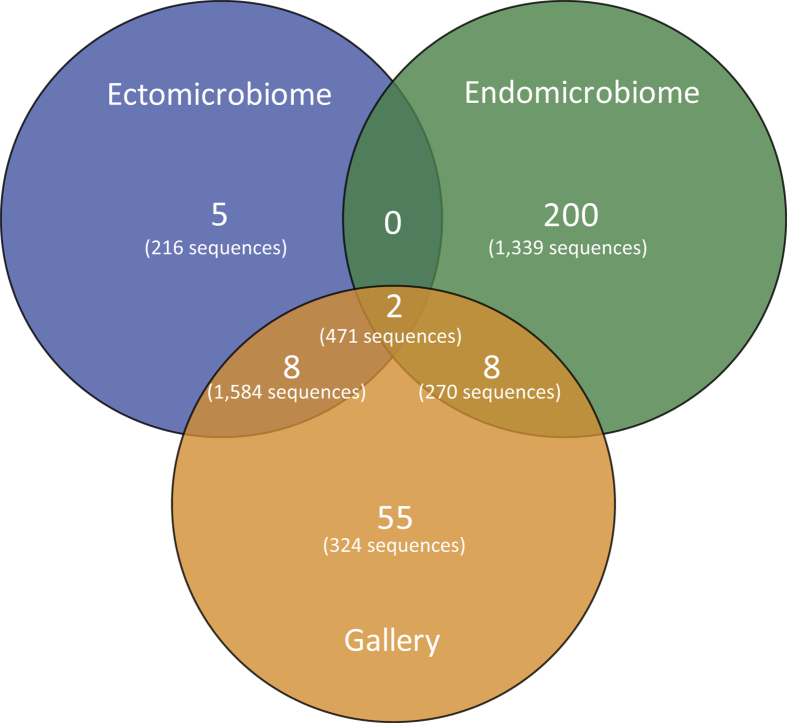
Venn diagram representing the distribution of the OTUs across the different environments. Only the high quality filtered sequences clustered at a 95% pairwise-identity threshold were used to generate the diagram. The number of OTUs and sequences is shown for each environment type.

A Venn diagram was also constructed to visualize the partition of the OTUs across the different environment types (Fig. 4). As mentioned before, more OTUs were found associated with the endomicrobiome. Some OTUs are shared between the different niches, as observed in the heat map (Fig. 3). One important observation is that there is no OTU shared only between the ectomicrobiome and endomicrobiome, further highlighting the difference in bacterial communities between the surface of the cuticle and the interior of the insect body. Moreover, few OTUs are shared between the ectomicrobiome and the microbiome of the galleries, as well as between the endomicrobiome and the microbiome of the galleries.

**Figure 5:**
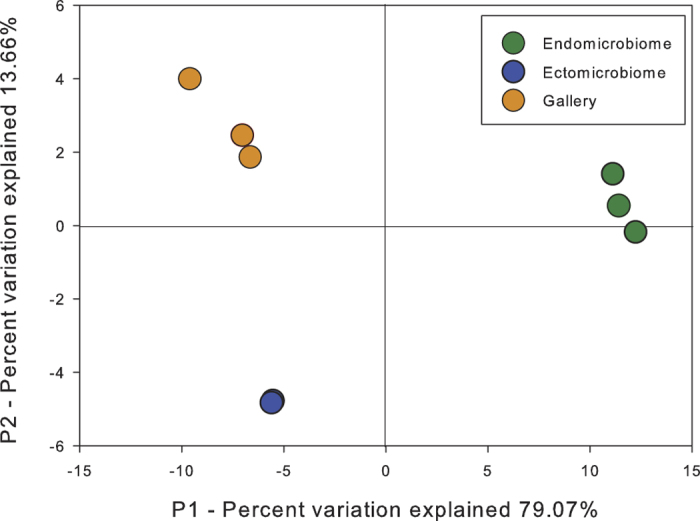
Principal coordinates analysis (PCoA) of bacterial communities associated with *Dendroctonus simplex* using Fast UniFrac. The percentages of variation explained by each axis are shown in parentheses. Equal sampling depths were used for each sample.

To ascertain whether different bacterial communities were distributed along the axes of variation, a principal coordinate analysis (PCoA) was performed. Figure 5 shows how the different bacterial populations are distributed. The first axis (P1) explains 79.1% of the variation and clearly distinguishes the ectomicrobiome and the microbiome of the galleries from the endomicrobiome. However, this axis doesn’t differentiate the ectomicrobiome and the galleries. The second axis (P2) explains 13.7% of the variation and mostly influences the ectomicrobiome and the microbiome of the galleries. Together, these axes explain 92.7% of the variability observed between sampled environments. The PCoA analysis clearly distinguishes among the three environments, highlighting once again their specific divergence.

## Discussion

The aim of this work was to characterize the bacterial microbiome associated with the eastern larch beetle. We examined the diversity of the ecto- and endomicrobiome of *D. simplex* adults. And since the immediate surroundings in which the beetles live may also influence their microbiome, the microbial diversity of the galleries from which the insects were sampled was also investigated. Such microorganisms could have an impact on the eastern larch beetle’s microbiome^22^. To identify the widest bacterial diversity possible, the 16S rRNA was sequenced by pyrosequencing, using the universal primer pair 28F-519R to cover the variable V1-V3 regions, widely used for the identification of bacteria. The V2 region is one of the two regions that provides the most accurate taxonomic classification with the lowest error rate^34^. The rarefaction curves for these samples tend toward an asymptote, showing that the identified diversity was fully suitable for the aims of this study. However, a higher number of sequences would be necessary to detect all diversity associated with the endomicrobiome. It is noteworthy that the experiment was repeated the following year with insects sampled at the same location, and obtained results fully validated those presented here (unpublished).

In order to compare bacterial diversity between samples, diversity indexes were calculated (Table 1). The Shannon index lends importance to the rare OTUs and is sensitive to their abundance. In contrast, the Ace index does not give a greater importance to OTUs with fewer sequences and thus constitutes an appropriate diversity measure when the number of OTUs to analyze is small^41^. To better understand the diversity of the samples, both indexes were used. Shannon’s diversity index showed a significant difference between the three environments, highlighting the specific bacterial communities identified. However, Ace showed a significant difference between the endomicrobiome and the two other conditions, but not between the ectomicrobiome and the microbiome associated to galleries. These results may indicate that diversity observed between the ectomicrobiome and the microbiome of the galleries is influenced by the non-abundant OTUs. In the literature, a large number of bacteria have been isolated from gut of several insect species, revealing its diversity^3^,^8^,^42^,^43^. Because many bacteria are significant factors for cellulose metabolism and for the acquisition of various nutrients, bacterial diversity of endomicrobiome of insects should be more complex. Despite the fact that some bacteria associated to bark beetles were previously recovered in the oral secretions or mouthparts^15^,^44^, our results shown for the first time the presence of microbiome associated to the cuticle of the *D. simplex* adults. Some bark and ambrosia beetles possess mycangia, which are transport structures for symbiotic bacteria and fungi^45^. However, the eastern larch beetle is one of the bark beetle species without mycangia^45^. Thus, the associated bacteria may be carried on the cuticle of an insect, probably under the elytra (unpublished observations). The absence of bacterial transportation structure could explain the lower diversity related to the ectomicrobiome compare to that of the endomicrobiome of the eastern larch beetles.

*Pseudomonas* sp., *Rahnella* sp., *Serratia* sp., *Pantoea* sp. and *Erwinia* sp. have been previously detected and isolated from several *Dendroctonus* species^12^,^13^,^46^,^47^. Experiments have shown that some of these bacteria can reduce terpene concentrations^12^,^46^. A community metagenomic analysis of the mountain pine beetle (*D. ponderosae*) revealed that the majority of genes responsible for terpene degradation were associated with bacteria from the genera *Pseudomonas* and *Rahnella*^13^. Furthermore, *Pseudomonas* species produce a large variety of antifungal metabolites^48^,^49^,^50^. Because the antifungal production is one of the significant roles played by symbiotic bacteria across the *Dendroctonus* genus, it is possible that *Pseudomonas* sp. associated with *D. simplex* are also involved in that function^16^,^17^. Consequently, terpene degradation and the production of antifungal metabolites might explain the presence of those bacteria to the *D. simplex* adult ectomicrobiome. Abundant bacteria found associated to the endomicrobiome were almost all Gammaproteobacteria, with also Firmicutes. In the phylogenetic tree (Fig. 3), the majority of endomicrobial OTUs are divergent from known bacterial species present in Genbank. These well-delineated OTUs might belong to novel bacterial genera or/and species. Enterobacteriaceae bacteria have been already found in beetle’s gut, where they were implicated in nitrogen-fixing processes and cellulolytic activities^7^,^8^,^9^,^43^. *Pseudoxanthomonas*, *Gilvibacter*, *Terriglobus* and *Raoultella* are also associated with the gut of several *Dendroctonus* species^8^,^10^. It is thus possible that some of these apparently new identified bacteria are involved in important nutritional roles. No shared bacterial genus was observed between the ectomicrobiome and endomicrobiome of *D. simplex*, which suggests that these distinct populations play exclusive roles, supporting a symbiotic implication.

Data observed are supported by the diversity analysis performed with mothur and Fast UniFrac. The environmental cluster regrouped the ectomicrobiome and the galleries sample closer together, thus clearly distinguishing the endomicrobiome. The Fast UniFrac analysis is based on the phylogeny and the relative abundance of the identified OTUs in each environment. Clearly, bacteria found on the surface of the insect cuticle and the interior of the insect body, are phylogenetically dissimilar, most probably because of environmental selection. The galleries and the ectomicrobiome cluster closer together, reflecting some overlap of the bacterial genera previously observed. Still, the PCoA analysis clearly distinguishes the three ecological niches (Fig. 5). The difference in the bacterial OTUs identified in the ectomicrobiome and the endomicrobiome was to be expected because of the difference in the environment they were recovered from. Surprisingly, the bacterial OTUs identified in the ectomicrobiome and the microbiome of the galleries harbor substantial differences, which, once again, reflect the high specificity of the bacterial communities identified in this study. This finding could indicate some selection of bacteria by the insect or a pressure from the environment. Altogether, our results indicate a specific organization between the bacterial communities. *D. simplex* also play a role as a vector of colonization of new ecological niches for microbes. Therefore, co-evolution may occur between the insect and its microbiome.

Finally, while significant bacterial diversity was found in the three niches investigated, a limited number of OTUs were predominant in each, with specific bacteria related to each of these ecological niches. Such a structured organization strongly suggests evolved specific interactions between *D. simplex* and its microbiome. Probably associated bacteria play different roles in each *D. simplex* environment, all of which may be complementary to the success of a beetle colonization attempt. The beetles used for this study were pioneers, as they were first to colonize the host tree. From a fitness perspective, these beetles should have carried the microorganisms conferring benefits related to initial host colonization.

This study is the first to report an association between the eastern larch beetle and a bacterial community. Moreover, it is the first to distinguish the ectomicrobiome and endomicrobiome of an insect and revealed significant differences. To get a complete picture of the microorganisms associated with *D. simplex*, investigation of the eukaryotic microbiome should be addressed. These findings will lead to the complete characterization of the eastern larch beetle symbiotic complex. Future directions will aim to isolate identified bacteria in order to investigate the functions in which they are involved.

## Additional Information

**How to cite this article**: Durand, A.-A. *et al*. Surveying the endomicrobiome and ectomicrobiome of bark beetles: The case of *Dendroctonus simplex*. *Sci. Rep*. **5**, 17190; doi: 10.1038/srep17190 (2015).

## Figures and Tables

**Table 1:** Diversity associated with the external surface and the interior of the eastern larch beetle body as well as the galleries.

Environment	Shannon (sd)	Ace (sd)
ECTO	1.267 (0.008)^a^	16.684 (4.464)^a^
GAL	2.240 (0.377)^b^	60.931 (32.298)^a^
ENDO	4.150 (0.296)^c^	148.895 (18.453)^b^

The abundance-base coverage estimator (Ace) and the Shannon diversity index were used to compare richness within the samples. Each of the samples contains 537 sequences to obtain equal sampling depths. All generated OTUs were used to calculate the diversity indexes. “Ecto”, ectomicrobiome; “Gal”, microbiome of the galleries; “Endo”, endomicrobiome; “sd”, standard deviation.
